# In the fight against HIV/AIDS: the arduous implementation of government-funded pre-exposure prophylaxis programme in Taiwan

**DOI:** 10.1136/sextrans-2023-055917

**Published:** 2024-04-04

**Authors:** Hsun Yin Huang, Jyun Rong Huang, Pei Chun Chan, Chia Chi Lee

**Affiliations:** 1 Division of Chronic Infectious Diseases, Taiwan Centers for Disease Control, Taipei, Taiwan; 2 Institute of Epidemiology and Preventive Medicine, College of Public Health, National Taiwan University, Taipei, Taiwan; 3 Division of Pediatric Infectious Diseases, Department of Pediatrics, National Taiwan University Hospital, Taipei, Taiwan

**Keywords:** HIV, HIV pre-exposure prophylaxis, HIV Seropositivity

## Abstract

**Introduction:**

The government-funded pre-exposure prophylaxis (PrEP) programme was targeted to those aged under 30 years or serodiscordant couples and implemented in September 2018–October 2020 in Taiwan. The study aimed to examine the effectiveness of the programme and the relationship between sexually transmitted disease (STD) and HIV seroconversion.

**Methods:**

This study was a retrospective cohort analysis with questionnaires designed for participants who joined the aforementioned programme in the PrEP-designated hospitals. The questionnaires included sociodemographic factors, sexual risk behaviours, number and types of sexual partners, and usage of narcotics filled in at the beginning of the programme and every 3 months. The McNemar test was used for the paired questionnaire analysis. The HIV seroconversion status among STD-notified patients nationwide was confirmed by using the data linkage method, followed up until October 2021 with stratification of PrEP programme participation or not.

**Results:**

The programme recruited 2155 people. 11 participants (0.5%) had seroconversion within the programme, while 26 (1.2%) had seroconversion after withdrawing from the programme. Overall, 1892 subjects with repeated questionnaires were included in the analysis for behaviour changes with median follow-up of 289 days. After joining the programme, 94.7% of them claimed that they had sexual behaviours: the rate of those who had condomless sex rose to 5.5% (p<0.001) and the rate of those who used narcotics decreased to 2% (p<0.001), compared with their response in the pre-questionnaire. Notably, the frequency of non-use of narcotics in recent 3 months increased from 16.9% to 38.4% in the pre-questionnaire and post-questionnaire responses, among the 177 who had claimed narcotics usage in recent 12 months (p=0.003). More HIV seroconversion was found among patients with STD who did not join the programme than those who joined the programme (8.7% vs 4.9%, p=0.031).

**Conclusions:**

The government-funded programme showed HIV case reduction and positive changes in health behaviours except for condomless sex which had increased prevalence. The reduction of HIV cases was also observed among people with STD. More resources should be allocated to the PrEP programme.

**WHAT IS ALREADY KNOWN ON THIS TOPIC**

- Daily pre-exposure prophylaxis (PrEP) is a preventive choice for people at risk of HIV infection with studies showing 44–96% reduction of HIV upon its use.
- However, there were limited implementation studies about how to implement PrEP policies or its effectiveness especially those run by the governments of Asian countries.

**WHAT THIS STUDY ADDS**

- According to the implementation experiences in Taiwan, to design the programme, allocating the resources to serodiscordant couples and the young population, who are at a socioeconomic disadvantage, was the key to run the programme smoothly.
- We have also observed that for those who were probably the riskiest population—patients with sexually transmitted disease—the HIV seroconversion rate was lower significantly among those who received PrEP than those who did not.

**HOW THIS STUDY MIGHT AFFECT RESEARCH, PRACTICE OR POLICY**

- The government-funded programme showed that PrEP alone could not effectively reduce the incidence of HIV but should be supported by healthier sexual behaviours such as less usage of narcotics.
- More resources should be considered to be allocated to the PrEP programme to turn the tide of HIV infection among high-risk populations.

## Introduction

Daily pre-exposure prophylaxis (PrEP) is a preventive choice for people at risk of HIV infection with studies showing 44–96% reduction of HIV upon its use.^1–3^ The WHO published the first guideline on PrEP in 2015 and recommended that countries implement the PrEP programme into national-level policy. However, as it has been difficult for conservative societies like Japan and South Korea to accept PrEP, there was a very small or even zero number of people using PrEP based on the official statistics as of October 2023.^4^ In Japan, there is not even an approved registration of emtricitabine/tenofovir disoproxil fumarate (TDF/FTC).^5^

The number of people living with HIV (PLHIV) was 34 544 by the end of 2021 in Taiwan, with 90% of PLHIV knowing their HIV status, 94% receiving antiretroviral therapy (ART) and 95% of those receiving ART achieving viral suppression.^6^ Sexual transmission in men who have sex with men is the leading transmission route (83.6%), followed by heterosexual contact (9.1%) and injecting drugs (1.7%).^7^ The introduction of the PrEP programme was recommended in a cross-ministerial meeting in 2015—the Committee for HIV Infection Control and Patient Rights Protection, held according to the HIV Infection Control and Patient Rights Protection Act.

The PrEP pilot programme kicked off on 15 November 2016^8 9^ aiming to enrol 1000 high-risk people. Not like other Asian countries with global funding or affordable generic drugs, there was only branded drug with patent protection in Taiwan. During that time, opinions about lesbian, gay, bisexual, transgender and queer (LGBTQ) rights continued to challenge the possibility of legislation of same-sex marriage in Taiwan. With the emergence of a potent force of public opinion, some conservative activists protested opposing same-sex marriage and claimed that HIV treatment would bring the influx of HIV-infected spouses and overwhelm the universal healthcare system. This misinformation also influenced the public’s opinion that PrEP should not be covered by government budget.^10–12^ The supreme court in Taiwan interpreted that the statutory ban on same-sex marriage in the Civil Code was ‘in violation of both the people’s freedom of marriage and the people’s right to equality as guaranteed by the Constitution’.^13 14^ The government temporarily paused the programme in September 2017 to avoid conflicts. Nevertheless, the preliminary result was exciting; only one person claimed he had terrible compliance and had HIV seroconversion among 302 participants,^15^ meaning the overall HIV seroconversion rate was 0.3%.

To appease the critics, resource allocation only to serodiscordant couples and the young population, taken as those with socioeconomic disadvantage, was adopted for the ‘stay low’ policy. Therefore, the programme was further extended to 18 cities (more than 80% of the country) in the third quarter of 2018 through a donation of 1000 person-years of Truvada for serodiscordant couples or those under 30 years. In cooperation with 31 hospitals (originally 5) and their PrEP managers, PrEP counselling was provided to high-risk people.^16^ To understand how effective the PrEP programme was, we performed an operational research to understand the changing behaviour, HIV seroconversion status and sexually transmitted disease (STD) status of those participants before and after enrolment in the PrEP programme.

## Methods

### Study design and setting

This study is a retrospective cohort analysis of longitudinal, routinely collected data from questionnaires among PrEP participants at a national level in September 2018–October 2020. 38 PrEP-designated hospitals were scattered around 18 cities in 2018, including 6 metropolitan cities whose newly reported HIV cases were more than the other 12 non-metropolitan cities, then gradually extended to 43 PrEP-designated hospitals distributed around 19 cities. Only three remote island cities did not join the PrEP programme in 2020. The HIV seroconversion status among patients with STD reported nationwide during the study period was confirmed by using the data linkage method.

### Process of enrolment

The PrEP programme was promoted through PrEP-designated hospitals, anonymous HIV testing hospitals or clinics, LGBTQ centres and health stations. Those willing to receive further consultation could call the PrEP-designated hospitals directly and make an appointment. However, for those who applied for free PrEP as serodiscordant couples, to ensure their identity, a referral sheet issued by the local health departments before making an appointment with PrEP-designated hospitals was needed.

During the appointment, participants needed to complete an individual-centred risk assessment on sociodemographic factors and sexual behaviours, including sex with condoms, usage of narcotics, and the number and types (regular or non-regular) of sexual partners (online supplemental appendix 1). Then, the PrEP manager would do the counselling and HIV testing, advise those with positive results to accept highly active ART and refer those with negative results to PrEP clinics for further information on the PrEP programme.

10.1136/sextrans-2023-055917.supp1 Supplementary data

The PrEP managers would ask participants with integrated needs for their life or mental problems like dementia or depression and refer them to related resources such as mental clinics and the like. Then they could receive 30 pills of free PrEP every month (up to 360 pills a year at most) and they needed to fill out questionnaires every 3 months and receive HIV testing. Loss to follow-up after 120 days of not returning would lead to being dropped out of the programme. Most frequent encountered reasons for dropout from the PrEP programme were seroconversion, intolerable side effects or events like breaking up with partners with HIV.

### Data sources and extraction

To understand the relationship between STD, PrEP programme and HIV seroconversion, HIV seroconversion status among patients with notified STD nationwide during the study period, stratified according to PrEP participation or not, was analysed. We cross-linked the national identification number of the participants in the programme to the notifiable disease reporting system. The definition of seroconversion of active syphilis, HIV, acute hepatitis A, B or C, and gonorrhoea was listed on the website of Taiwan Centers for Disease Control.^17^ All the participants were followed up until October 2021 for HIV seroconversion.

### Statistical analysis

For analysis of sex and health behaviours, comparing the answers to the last questionnaire the participants filled out in the programme with the first questionnaire before they joined the programme was done to see the changes. PrEP participants without at least one subsequent visit were excluded from the analysis. Since the study subjects may not represent the population and non-parametric paired nominal nature data, the McNemar test was used for the paired questionnaire analysis. As for the comparison of HIV seroconversion rate among patients with STD stratified by PrEP programme participation or not, Χ^2^ test was used for analysis. SPSS V.22 and Excel V.2016 were used as the statistical software.

## Results

The programme recruited 2155 people during the study period, including 1552 people for the subset of age under 30 years and 603 people as serodiscordant couples. As for the characteristics of participants, 97.4% were male and 81.2% lived in the metropolitan areas. The mean age was 28 years and 86.9% had a high school or higher educational level.

For active syphilis, 375 people (17.4%) had notification history at enrolment; 32 persons 1.5%) observed a fourfold change in titre and were notified again when they were in the programme; and 115 (5.3%) people were notified of their syphilis for the first time within the programme. A total of 37 participants (1.7%) tested HIV positive: 11 participants (0.5%) were seroconverted within the programme, while 26 (1.2%) were seroconverted after withdrawing from the programme (table 1).

**Table 1:** Distribution of sociodemographic characteristics of participants in the pre-exposure prophylaxis programme

Characteristics	Total	
	N	%
Total	2155	100
Sex		
Male	2099	97.4
Female	56	2.6
Residence		
Metropolitan areas	1750	81.2
Non-metropolitan areas	405	18.8
Age		
Mean (±SD)	28 (±5.7)	
Median (Q1–Q3)	27(24−30)	
Education		
High school or below	282	13.1
University	1533	71.2
Master’s degree or above	339	15.7
Active syphilis		
Negative or no testing result	1665	77.3
Positive	490	22.7
Before the programme	375	17.4
Within the programme	147	6.8
A fourfold change in titre	32	1.5
Seroconversion	115	5.3
HIV		
Remained negative	2118	98.3
Conversion to positive	37	1.7
Within the programme	11	0.5
After the programme	26	1.2

Among the 2155 participants, 263 did not return for the second visit, so 1892 subjects were included in the final analysis for behavioural changes. The average and median duration of staying in the programme were 276 and 289 days, respectively. The 263 participants who did not return lived in non-metropolitan areas, had lower education level and had a lower number of non-regular sex partners in recent 3 months, compared with those who returned regularly. Since they no longer took medications, they also had higher HIV conversion rate (data not shown). Table 2 shows the similarities and differences in the pre-questionnaire and post-questionnaire responses for the 1892 participants. On the aspect of frequency of narcotics use in recent 3 months, among the 177 who had claimed narcotics usage in recent 12 months in pre-questionnaire or post-questionnaire responses, only 16.9% of them claimed that they did not take any drug in recent 3 months, 45.8% claimed once a month or less, 21.5% two to three times a month, 13.6% at least once a week and 2.3% at least once a day. Among the 79 who filled in chemsex information in pre-questionnaire or post-questionnaire responses, 6.3% claimed that they never had substance use before or during sexual activities, and 53.2% claimed that they always or usually had chemsex. Among those who had chemsex, 33.3% claimed that they never use condoms when having sex, 42.3% claimed that they seldom use them and only 6.4% claimed that they always use condoms during chemsex. 84.8% of the participants said they did not currently attend mental clinics.

**Table 2:** Changes in sexual risk behaviour before and after joining a PrEP programme

Items	Pre-questionnaire	Post-questionnaire	McNemar test
			P value
Sex in recent 12 months			
No	33 (1.7%)	101 (5.3%)	<0.0001
Yes	1859 (98.3%)	1791 (94.7%)	
Condomless sexual behaviours in recent 12 months			
No	1566 (82.8%)	1462 (77.3%)	<0.0001
Yes	326 (17.2%)	430 (22.7%)	
Number of regular sex partners in recent 3 months			
≤1	1360 (71.9%)	1388 (73.4%)	0.231
≥2	532 (28.1%)	504 (26.6%)	
Number of non-regular sex partners in recent 3 months			
≤1	677 (35.8%)	881 (46.6%)	<0.0001
≥2	1215 (64.2%)	1011 (53.4%)	
Narcotics usage in recent 12 months			
No	1745 (92.2%)	1783 (94.2%)	<0.0001
Yes*	147 (7.8%)	109 (5.8%)	
Amphetamine (both slam or inhalation)	98 (55.4%)	89 (50.3%)	
Amphetamine (inhalation only)	95 (53.7%)	80 (45.2%)	
MDMA	35 (19.8%)	27 (15.3%)	
Marijuana	31 (17.5%)	17 (9.6%)	
Amphetamine (slam only)	19 (10.7%)	28 (15.8%)	
GHB	21 (11.9%)	17 (9.6%)	
Ketamine	11 (6.2%)	5 (2.8%)	
LSD	5 (2.8%)	3 (1.7%)	
Erimine	4 (2.3%)	2 (1.1%)	
5-MeO	4 (2.3%)	1 (0.6%)	
Crack cocaine	3 (1.7%)	1 (0.6%)	
Heroin	2 (1.1%)	1 (0.6%)	
FM2	2 (1.1%)	2 (1.1%)	
Mephedrone	1 (0.6%)	0 (0.0%)	
Others	13 (7.3%)	3 (1.7%)	
Frequency of usage of narcotics in recent 3 months			
No	30 (16.9%)	68 (38.4%)	0.003
Yes*	147 (83.1%)	109 (61.6%)	
Once a month or less	81 (45.8%)	67 (37.9%)	
2–3 times a month	38 (21.5%)	26 (14.7%)	
At least once a week	24 (13.6%)	13 (7.3%)	
At least once a day	4 (2.3%)	3 (1.7%)	
Chemsex			
No	5 (6.3%)	5 (6.3%)	0.162
Yes†	74 (93.7%)	74 (93.7%)	
≤30%	13 (16.5%)	10 (12.7%)	
30~50%	12 (15.2%)	21 (26.6%)	
50~80%	7 (8.9%)	10 (12.7%)	
≥80%	18 (22.8%)	15 (19.0%)	
Always	24 (30.4%)	18 (22.8%)	
Usage of condoms when having chemsex			
No	26 (33.3%)	37 (47.4%)	0.084
Yes	52 (66.7%)	41 (52.6%)	
Seldom	33 (42.3%)	31 (39.7%)	
Usually	14 (17.9%)	7 (9.0%)	
Always	5 (6.4%)	3 (3.8%)	
Under treatment in mental clinics			
No	67 (84.8%)	62 (78.5%)	0.302
Yes‡	12 (15.2%)	17 (21.5%)	

*The average and median duration (the period between pre-questionnaire and post-questionnaire response) were 276 and 289 days, and the denominator was 177 for those who had claimed narcotics usage in recent 12 months in the pre-questionnaire or post-questionnaire response.

†The denominator was 79 for those who had claimed chemsex in the pre-questionnaire or post- questionnaire response.

‡The denominator was 79 for those who had claimed usage of narcotics and under treatment in mental clinics in the pre-questionnaire or post-questionnaire response.

PrEP, pre-exposure prophylaxis.

After joining the programme, 94.7% of them claimed that they had sex: the rate of those who had condomless sex rose to 5.5% (p<0.001) and the rate of those who used narcotics decreased to 2% (p<0.001), compared with their response in the pre-questionnaire. The percentage of those with more than two non-regular sexual partners decreased from 64.2% to 53.4% (p<0.001). 93.7% of them who had chemsex in the beginning still had chemsex after, and among them, the percentage of those who never used condoms when having sex increased for about 14.1% and the percentage of those attending mental clinics increased for about 6.3%, without statistical significance. Notably, the rate of people who did not use narcotics in recent 3 months increased from 16.9% to 38.4% in the pre-questionnaire and post-questionnaire responses, among the 177 who had claimed narcotics usage in recent 12 months in the post-questionnaire response (p=0.003). The decreasing trend could be observed in variable stratification of frequency of narcotics usage.

Our analysis showed that after joining the programme, the rate of condomless sex behaviour increases, while the number of non-regular sexual partners was fewer than the number at baseline. To find out which subgroup may change towards safer sexual behaviour, we investigated the distribution of condomless sexual behaviour in recent 12 months stratified by the number of non-regular partners (table 3). Among two groups, including those who claimed they have fewer non-regular sex partners in both pre-questionnaire and post-questionnaire responses, or changed to no more than two sex partners in the post-questionnaire response, the percentage of those who engaged in condomless sexual behaviour in recent 12 months had decreased, which was not in accordance with the total trend of increasing rate of condomless sexual behaviour.

**Table 3:** The distribution of condomless sexual behaviour in recent 12 months among groups with changing numbers of non-regular sex partners

Number of non-regular sex partners	Condomless sexual behaviour in recent 12 months		McNemar test p value
	Pre-questionnaire	Post-questionnaire	
**Subgroups**	1892		
Pre/post ≤1 non-regular sex partner			
Total	528 (27.9%)		<0.0001
No	129 (24.4%)	177 (33.5%)	
Yes	399 (75.6%)	351 (66.5%)	
Pre ≤1 non-regular sex partner, post ≥2 non-regular sex partners			
Total	149 (7.9%)		0.627
No	29 (19.5%)	25 (16.8%)	
Yes	120 (80.5%)	124 (83.2%)	
Pre ≥2 non-regular sex partners, post ≤1 non-regular sex partner			
Total	353 (18.7%)		0.0008
No	54 (15.3%)	91 (25.8%)	
Yes	299 (84.7%)	262 (74.2%)	
Pre/post ≥2 non-regular sex partners			
Total	862 (45.6%)		0.096
No	114 (13.2%)	137 (15.9%)	
Yes	748 (86.8%)	725 (84.1%)	

In the study period, there were 9784 reported patients with STD nationwide (table 4). Among the 265 (2.7%) patients with STD who joined the programme, 13 (4.9%) had HIV seroconversion. On the contrary, a higher proportion of patients with STD who did not join the programme had HIV seroconversion compared with those who joined the programme (8.7% vs 4.9%, p=0.031).

**Table 4:** HIV seroconversion status among high-risk population (notified with sexually transmitted diseases) in September 2018–October 2020

Joined the PrEP programme	Total	HIV seroconversion		P value*
		Yes	No	
Yes	265 (2.7%)	13 (4.9%)	252 (95.1%)	0.031
No	9519 (97.3%)	824 (8.7%)	8695 (91.3%)	
Total	9784 (100%)	837 (8.6%)	8947 (91.4%)	

*X^2^ test.

PrEP, pre-exposure prophylaxis.

## Discussion

The implementation of PrEP programme in Taiwan was not as smooth as the government anticipated, so evaluation of the outcome and effectiveness was inevitably necessary. Since the HIV seroconversion rate after withdrawing from the programme was higher than within the programme (1.2% vs 0.5%), protecting key population including people aged under 30 years and serodiscordant couples from HIV infection was evidenced. Real-world data supported that PrEP indeed accelerated the reduction of HIV infection.^18 19^ The HIV seroconversion rate among those who had been reported to have STD including active syphilis, gonorrhoea and acute hepatitis A, B or C was 8.7%, significantly higher than those who joined the PrEP programme (4.9%), showing that the PrEP programme could prevent HIV infection significantly. To our knowledge, there was no study related to retrospective comparisons of HIV among those who got STD infection already.

We also observed the decreasing trend of the number of non-regular sexual partners before and after participation in the PrEP programme. Other studies also showed no change or decreasing trend for those who took PrEP.^20 21^ We may infer that aside from the effect of the PrEP medication itself, the programme indirectly provided participants health information on safe sex and regular HIV testing, which further helped them to focus on their behaviour.

In Taiwan, the predominant narcotics used for chemsex among key population were amphetamine (including slam), MDMA (ecstasy) and marijuana—contrary to recent studies observed in England,^22^ Canada^23^ and the Netherlands,^24^ where ecstasy, GHB and cocaine were the primary drugs used. However, the usage of amphetamine has been increasing in the Netherlands and Switzerland since 2007 or 2008 until 2017 or 2018.^25 26^ In most Asian countries in the past decade, the predominant drug used during chemsex was still amphetamine.^27^ It is hard to conclude on the different types of narcotics used since the usage of drugs related to chemsex may vary according to subculture, accessibility and other socially constructed factors.^28^

In general, the increasing trend of risk compensation behaviour, including condomless sexual behaviour, was observed in the study, which was not found in most of the earlier studies,^29^ but has been found in recent studies.^30^ The reason for more risk compensation behaviour we observed in the study may be due to the fact that PrEP users have more confidence in the effectiveness of PrEP. Decreasing use of condoms after taking PrEP may also reflect their belief that taking PrEP without condoms made them feel safe to seek more emotional intimacy and closeness^31^ and lower their anxiety.^32^ However, among those maintaining one or fewer non-regular sex partners or decreasing the number of non-regular sex partners, the decreasing of rate of condomless sexual behaviour was observed in this study. A study in the USA showed that the mean number of sex partners was decreasing and the mean number of condomless anal sex episodes was relatively stable^33^ after PrEP use. In the review, most articles found that it was much more difficult for non-regular partners to discuss relative intimate issues like wearing condoms.^34 35^ The inconsistent results of decreasing or increasing rate of condomless sexual behaviour we observed worldwide may be brought by different subculture, behaviour and psychosocial factors; thus, more qualitative studies may be needed to explore why this phenomenon occurred in Taiwan.

Through the PrEP programme, more participants claimed that they received mental health therapy, although not reaching statistical significance. Few studies used the factor ‘attending mental clinics’ as an outcome for evaluating the PrEP programme. However, with the increasing trend of chemsex, it was an important strategy to engage key population, to be supported by professional teams from constant harm. In our programme, 7.8% of participants had claimed that they had been involved with narcotics in the recent year. Offering a transfer fee for the case manager could incentivise them to convince patients to receive the therapy. The downward trends of chemsex were also observed after taking PrEP in Canada^23^ and the UK,^30^ although we did not observe the same trend in our study.

There were some limitations to the study. The first one is the healthy worker effect,^36^ which means that those who stayed in the programme are those who put more efforts over their health. On the contrary, those with worse compliance due to worse health status or deprived socioeconomic status cannot return regularly and thus would be excluded from our analysis. The second limitation is that recall bias existed due to self-reported questionnaires. Also, for answering sensitive and personal questions, the participants may not provide the factual situations due to privacy violation, especially when the PrEP manager would review them as an indicator.

## Conclusions

The lower seroconversion rate of HIV infection among participants who continued with the PrEP programme compared with those who withdrew from the programme indicated that the programme and PrEP managers played critical roles in eliminating HIV infection in Taiwan. As the risk compensation behaviour was observed, the rate of those who used narcotics decreased. The programme could be a window of resources (such as harm reduction for chemsex) needed by the high-risk population to protect themselves from HIV infection. Therefore, more resources should be allocated to the PrEP programme.

10.1136/sextrans-2023-055917.supp2 Abstract translation This web only file has been produced by the BMJ Publishing Group from an electronic file supplied by the author(s) and has not been edited for content.

## Data Availability

Data are available upon reasonable request.
