# Supplementary material for: In the fight against HIV/AIDS: the arduous implementation of government-funded pre-exposure prophylaxis programme in Taiwan
Source: Sex Transm Infect. 2024 Apr 4;100(4):216–21. doi: 10.1136/sextrans-2023-055917 (PMC11187399; doi:10.1136/sextrans-2023-055917)
Supplement: Supplementary data [file sextrans-2023-055917supp001.pdf]

Appendix 1. Questionnaires of enrollment / 3 months follow-up of PrEP program

Basic Information

Birth：_______(yyyy)_______(m)_________(d)

Residence：_____County/City

Sex：☐Male ☐Female ☐Trans

Orientation：☐Homosexual ☐Heterosexual ☐Bisexual

Education：☐Illiterate ☐Elementary school ☐Junior high school
☐High school/Vocational school ☐University ☐Master ☐Doctoral degree

Behavioral Assessment

- Do you have sexual behaviors in recent 12 months？
  ☐ Yes ☐No
- Do you have unprotected sexual behaviors in recent 12 months？
  ☐ Yes ☐No
- Have you have been diagnosed sexually transmitted diseases (ex. syphillis, gonorrhea, chlamydia, HPV)?
  ☐ Yes ☐No
- Have you been a sexual worker in recent 12 months？
  ☐ Yes ☐No
- Have you taken non-occupational post-exposure prophylaxis in recent 12 months？
  ☐ Yes ☐No
- Is your sexual partners the HIV infected ？
  ☐ Yes ☐No
  - If yes, does your partner (HIV positive) under the HAART treatment currently？
    ☐ Yes ☐No ☐ I don't know
  - If yes, does your partner (HIV positive) under the HAART treatment and with the virus suppression currently？
    ☐ Yes ☐No ☐ I don't know

Huang HY, *et al. Sex Transm Infect* 2024; 100:216–221. doi: 10.1136/sextrans-2023-055917

Narcotic Assessment

- Have you used narcotics in recent 12 months？
  ☐ Yes ☐No
  - If yes, what narcotics you have used in recent 3 months ？
    ☐Heroin ☐Crack cocaine ☐Amphetamine(inhalation only)
    ☐Amphetamine(slam only) ☐MDMA
    ☐Marijuana ☐Morphine ☐Secobarbital(Seconal)
    ☐Amobarbital(Amytal) ☐Methaqualone (Normi-Nox) ☐FM2
    ☐Ketamine ☐LSD ☐Erimine ☐Methadone (non-prescription)
    ☐GHB ☐RUSH ☐5-meo ☐Mephedrone ☐Other_________
  - If yes, what is the frequency of using narcotics in recent 3 months？
    ☐Once or below a month ☐2-3 times a month
    ☐Once a week ☐2-3 times a week
    ☐4-6 times a week ☐Once a day
    ☐2-3 times a day ☐4 or more times a day
  - If yes, where have you used narcotics？
    ☐At home ☐Home-party ☐Night clubs
    ☐Other entertainment venues ☐Other
  - If yes, what is the frequency of chemsex？
    ☐Always ☐≧80% ☐50~80% ☐30~50% ☐≦30% ☐Never
  - If yes, do you use condoms when you have chemsex？
    ☐Always ☐Usually ☐Seldom ☐Never
  - If yes, what is the reason why you did not use condoms when you have chemsex？
    ☐Too dizzy to use ☐My partners refuse to use ☐It's not necessary
    ☐Not available ☐Uncomfortable ☐Other_______________
- Among your friends, do you know that they have used narcotics in recent 12 months？
  ☐ Yes ☐No
- Among your friends, how many friends do you know that they have used narcotics in recent 12 months？
  ___ of 10 friends

- Are you under the treatment of mental clinics？
  ☐Yes ☐No

Risk-compensation behaviors Assessment

- What is the possibility do you think that you will get the HIV infection？
  ☐Impossible ☐Unlikely ☐Possible ☐Probable ☐Certain
- What is the number of regular sexual partners in recent 3 months?
  ☐0 ☐1 ☐2 ☐3 ☐4 ☐5 ☐6 ☐7 ☐8 ☐9 ☐10 or above
- What is the number of regular sexual partners in recent 3 months?
  ☐0 ☐1 ☐2 ☐3 ☐4 ☐5 ☐6 ☐7 ☐8 ☐9 ☐10 or above
- What is the frequency of using condoms when having sex in recent 3 months?
  ☐0% ☐10% ☐20% ☐30% ☐40% ☐50% ☐60% ☐70% ☐80% ☐90% ☐100%

Huang HY, *et al. Sex Transm Infect* 2024; 100:216–221. doi: 10.1136/sextrans-2023-055917
