# Supplementary material for: In the fight against HIV/AIDS: the arduous implementation of government-funded pre-exposure prophylaxis programme in Taiwan
Source: Sex Transm Infect. 2024 Apr 4;100(4):216–21. doi: 10.1136/sextrans-2023-055917 (PMC11187399; doi:10.1136/sextrans-2023-055917)
Supplement: Abstract translation [file sextrans-2023-055917supp002.pdf]

中文摘要

愛滋戰役-披荊斬棘的臺灣公費暴露愛滋病毒前預防性投藥（PrEP）計畫

背景介紹

臺灣公費暴露愛滋病毒前預防性投藥（PrEP）計畫補助 30 歲以下的年輕族群或愛滋病毒感染者的陰性配偶或伴侶，本研究為檢視計畫成效以及與性傳染病患者相較其感染愛滋病毒陽轉率，對於 2018 年 9 月至 2020 年 10 月計畫實施期間的民眾進行分析。

方法

本研究為一世代追蹤研究，民眾加入計畫及加入計畫每 3 個月回診領藥之際需填寫風險評估問卷，題目包括社會人口學變項、性行為、性伴侶數目與型態、是否使用成癮性物質等，並使用 McNemar 檢定進行風險評估問卷前後測的分析。全國性病患者的愛滋病毒陽轉狀態，則是使用資料勾稽的方式，針對否參加 PrEP 計畫，來進行分層分析，追蹤期限直至 2021 年 10 月。

結果

本研究計有 2,155 位民眾納入分析， 計有 11 名參與者（0.5%）於計畫內通報愛滋病毒感染，而有 26 名（1.2%）則於退出計畫後通報感染愛滋病毒。整體而

言，1,892 位民眾於計畫中有填列 2 次以上問卷方納入前後測分析，前後測期間中位數為 289 天。 針對加入民眾於計畫中最後一次與第一次填列的風險評估問卷進行分析，加入計畫後，無套性行為的比例上升至 5.5%（p <0.001），有 2%的民眾減少使用成癮性物質的頻率（p <0.001）。值得注意的是，於近 12 個月內聲稱有使用成癮性物質的 177 人中，最近 3 個月未使用成癮性物質的頻率從 16.9%增加到 38.4%（p=0.003）。且 與參加計畫的性病患者相比，未參加計畫的性病患者，HIV 陽轉率較高（8.7% vs. 4.9%，p=0.031）。

結論

參加公費 PrEP 計畫有效地減少愛滋病毒感染，除了無套性行為略增加，對其它健康行為產生了正面的影響。對愛滋感染的下降，還在性病患者中觀察到。建議應投入更多防疫資源於 PrEP 計畫。
